# Supplementary material for: Cold-Active Starch-Degrading Enzymes from a Cold and Alkaline Greenland Environment: Role of Ca2+ Ions and Conformational Dynamics in Psychrophilicity
Source: Biomolecules. 2025 Mar 14;15(3):415. doi: 10.3390/biom15030415 (PMC11940188; doi:10.3390/biom15030415)
Supplement: Supplementary file 1 [file biomolecules-15-00415-s001.zip › biomolecules-3506041-supplementary.pdf]

Supplementary Information

Cold-active starch degrading enzymes from a cold and alkaline environment in Greenland: impact of oligomerization on stability and activity

Malthe Kjær Bendtsen, Jan Stanislaw Nowak, Marcos López Hernández, Pedro Paiva, Pedro Ferreira, Francesco Bisiak, Jan Skov Pedersen, Lars Haastrup Pedersen, Ditlev E. Brodersen, Athanasios Zervas, Pedro A. Fernandes, Maria Joao Ramos, Peter Stougaard, Mariane Schmidt Thøgersen and Daniel Erik Otzen

Figure S1a

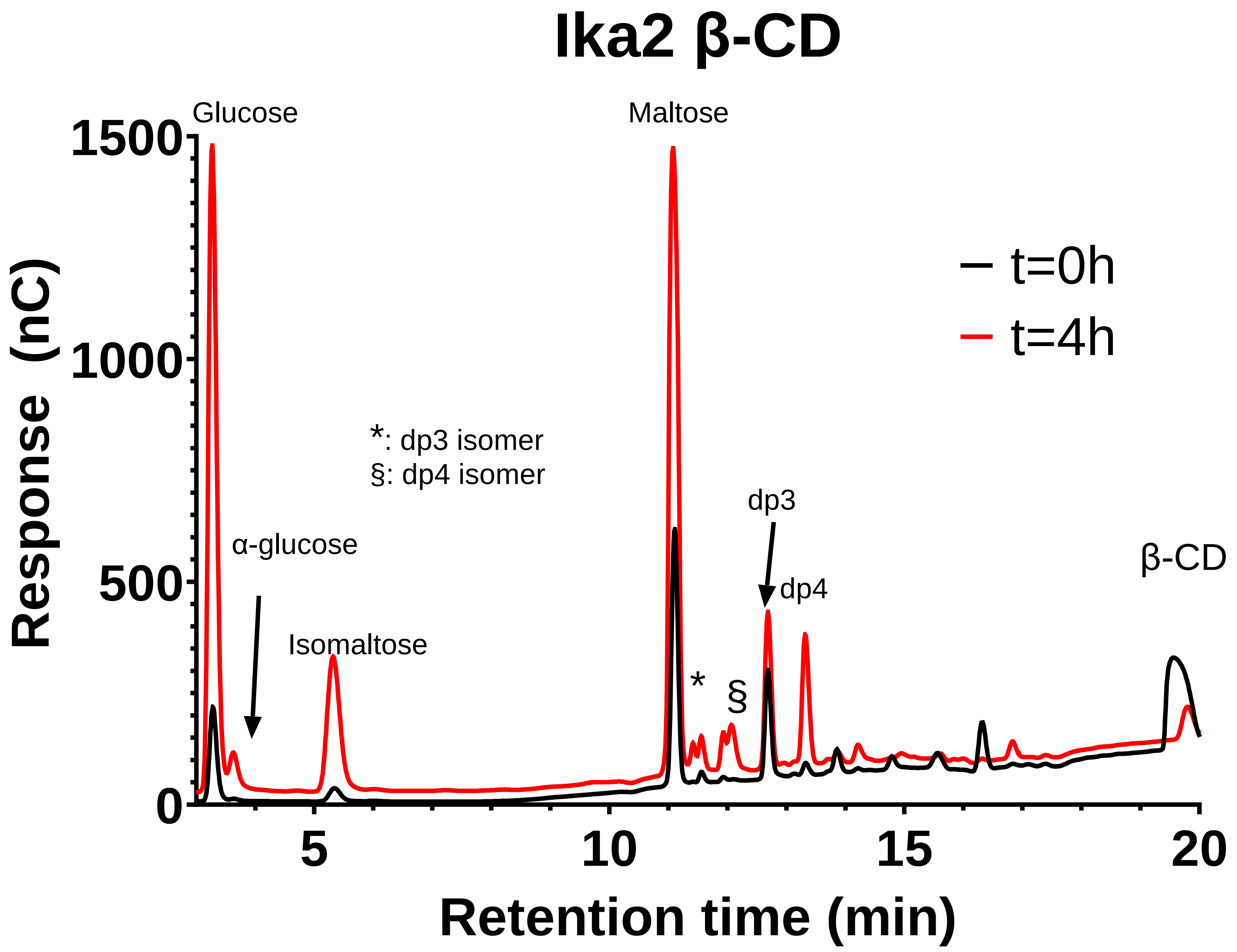
Ika2 β-CD
Glucose
Maltose
1500
1000
500
0
Response (nC)
t=0h
t=4h
*: dp3 isomer
§: dp4 isomer
α-glucose
Isomaltose
dp3
dp4
*
§
β-CD
5
10
15
20
Retention time (min)

Figure S1b

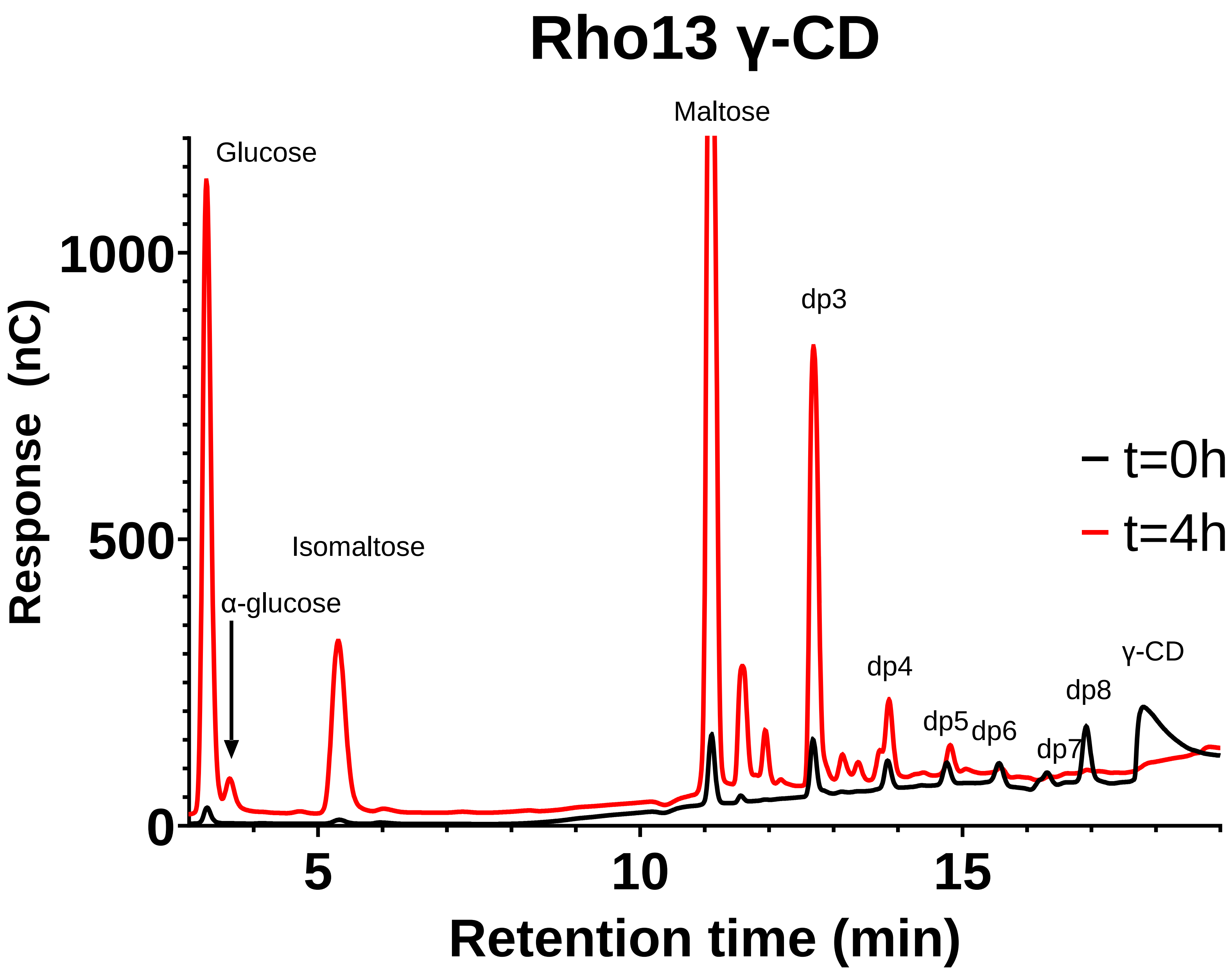

(See legend to **Fig. S1a**)

Figure S1c

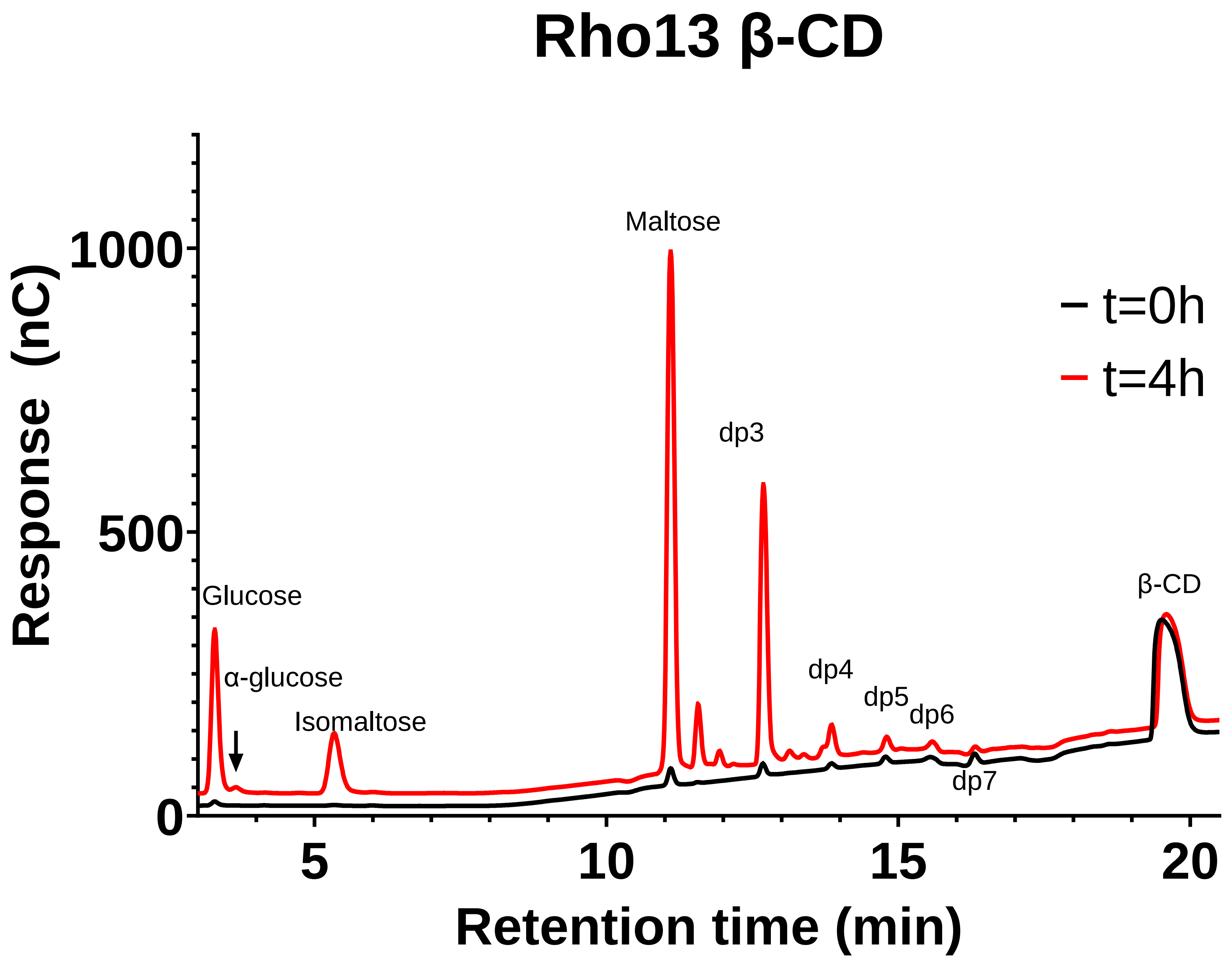

(See legend to **Fig. S1a**)

Figure S1d

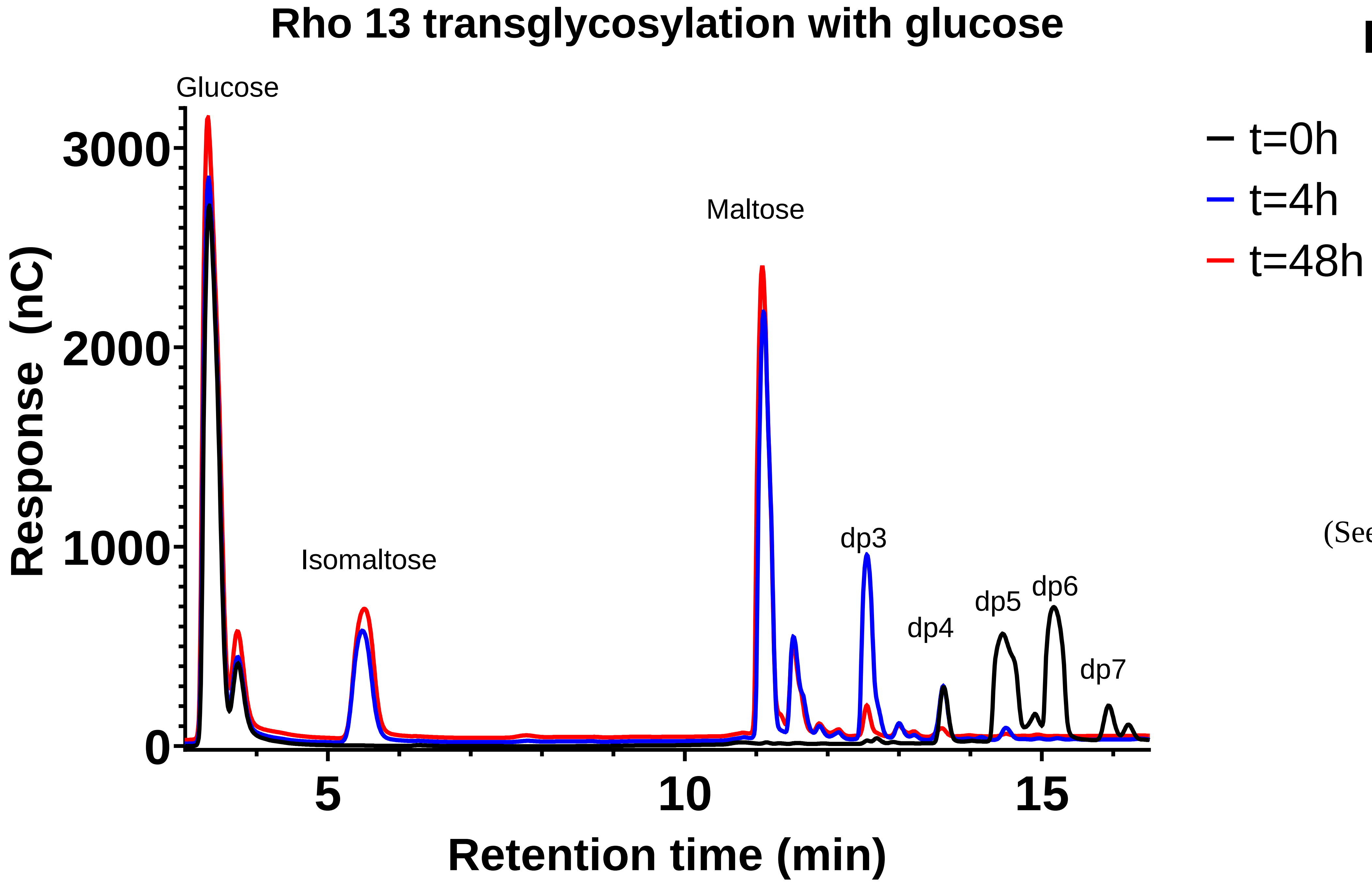

(See legend to **Fig. S1a**)

Figure S1e

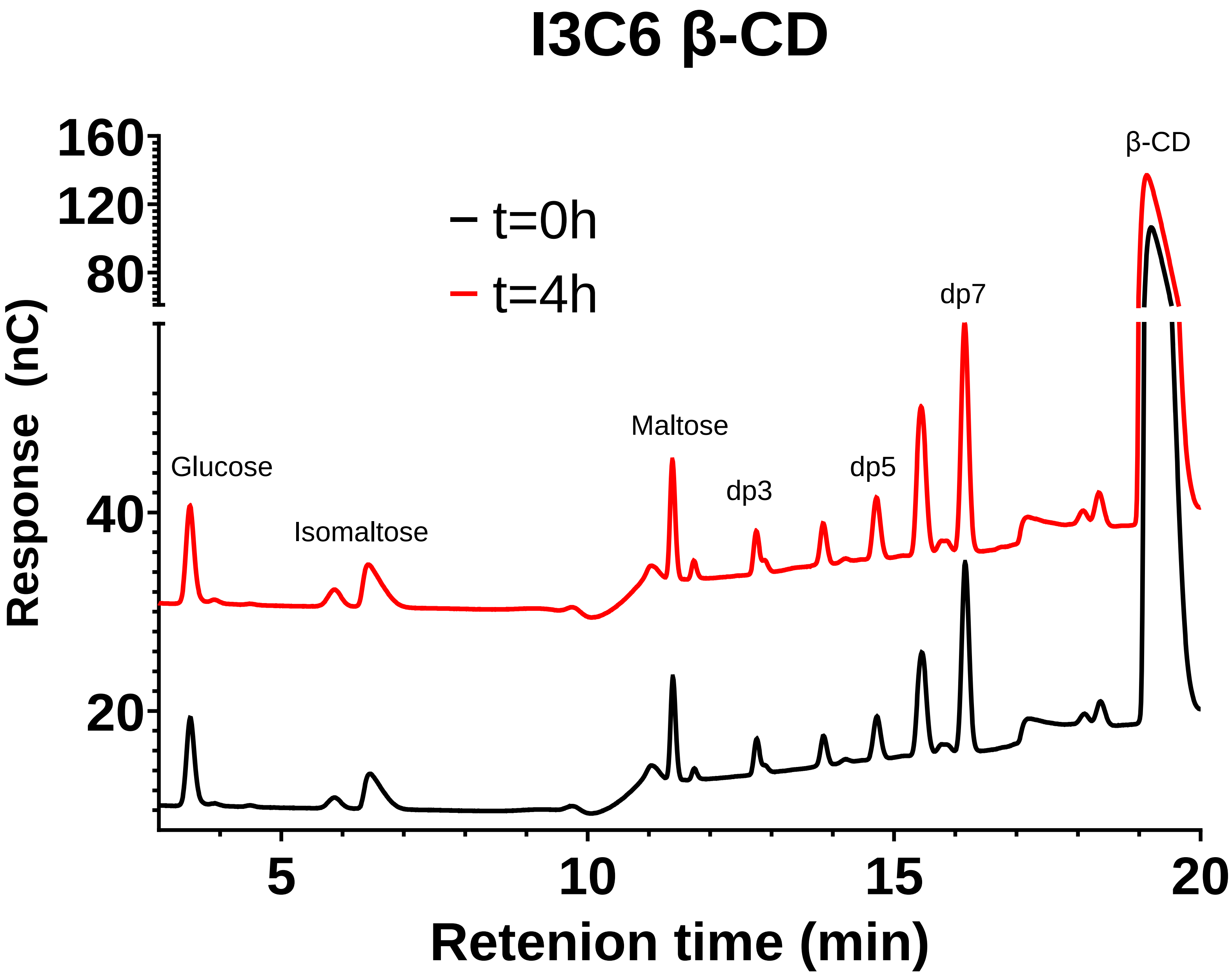

(See legend to **Fig. S1a**)

Figure S1f

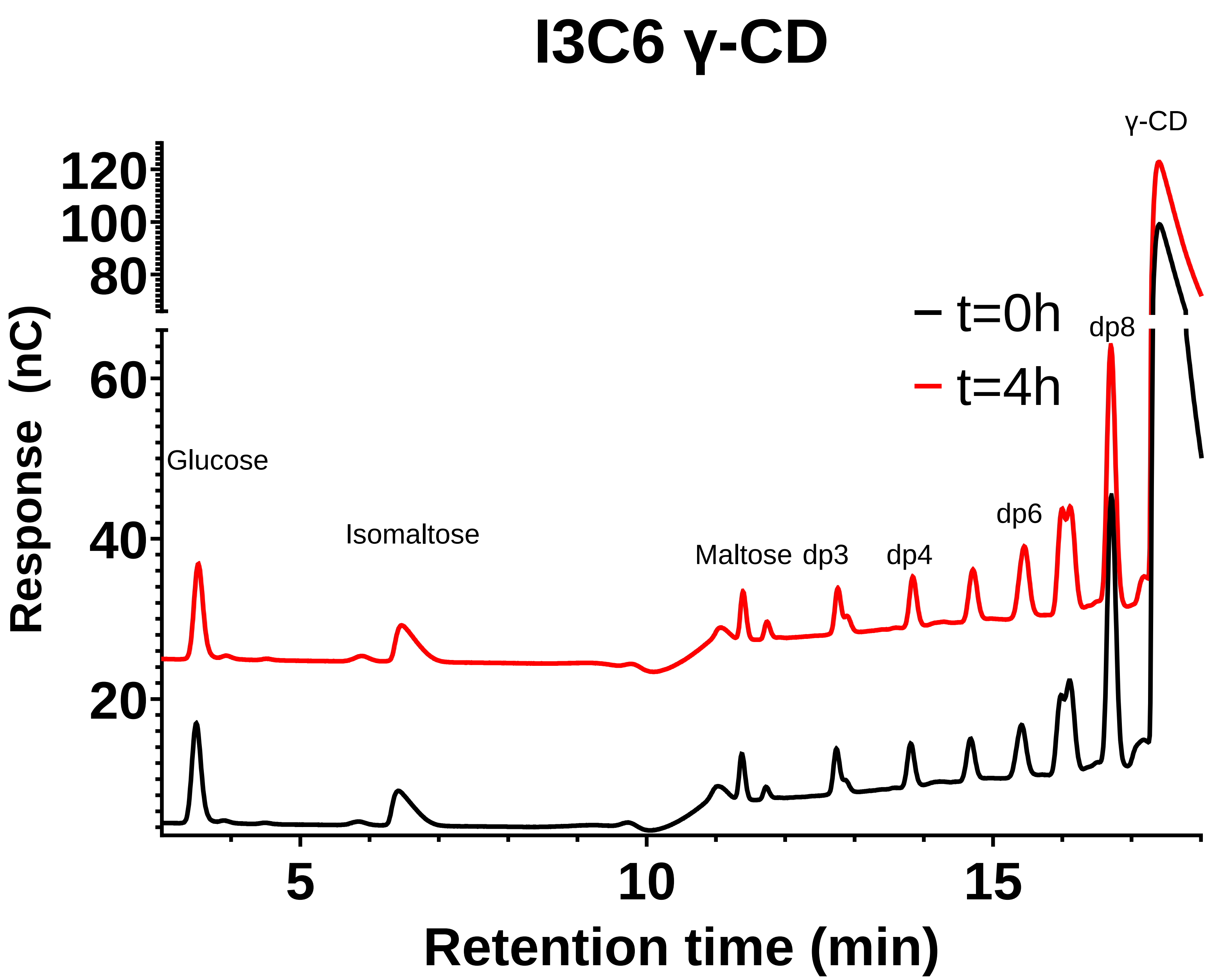

(See legend to **Fig. S1a**)

Figure S1g

I3C6 transglycosylation with glucose

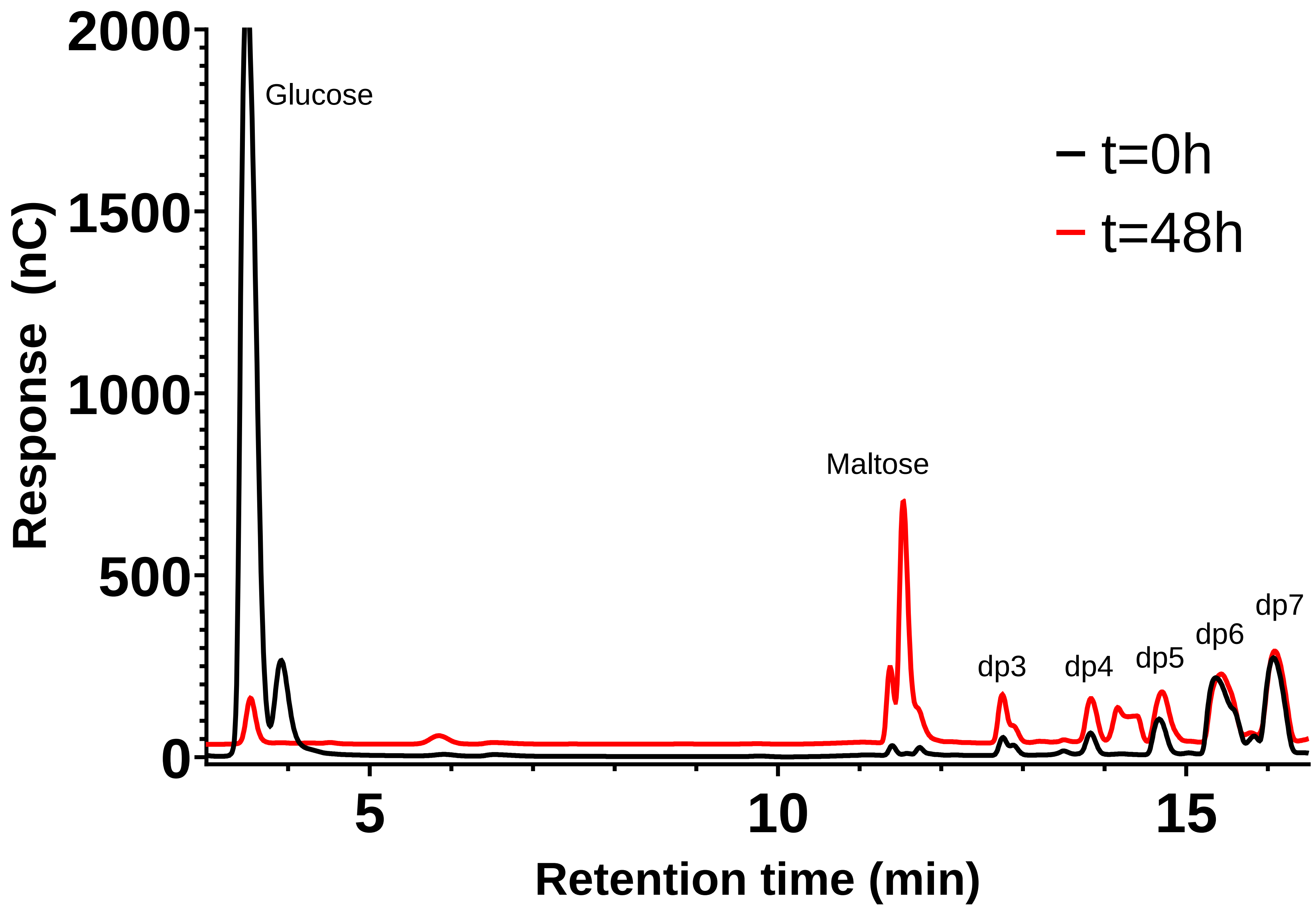

(See legend to **Fig. S1a**)

I3C6 transglycosylation with fucose

Figure S1h

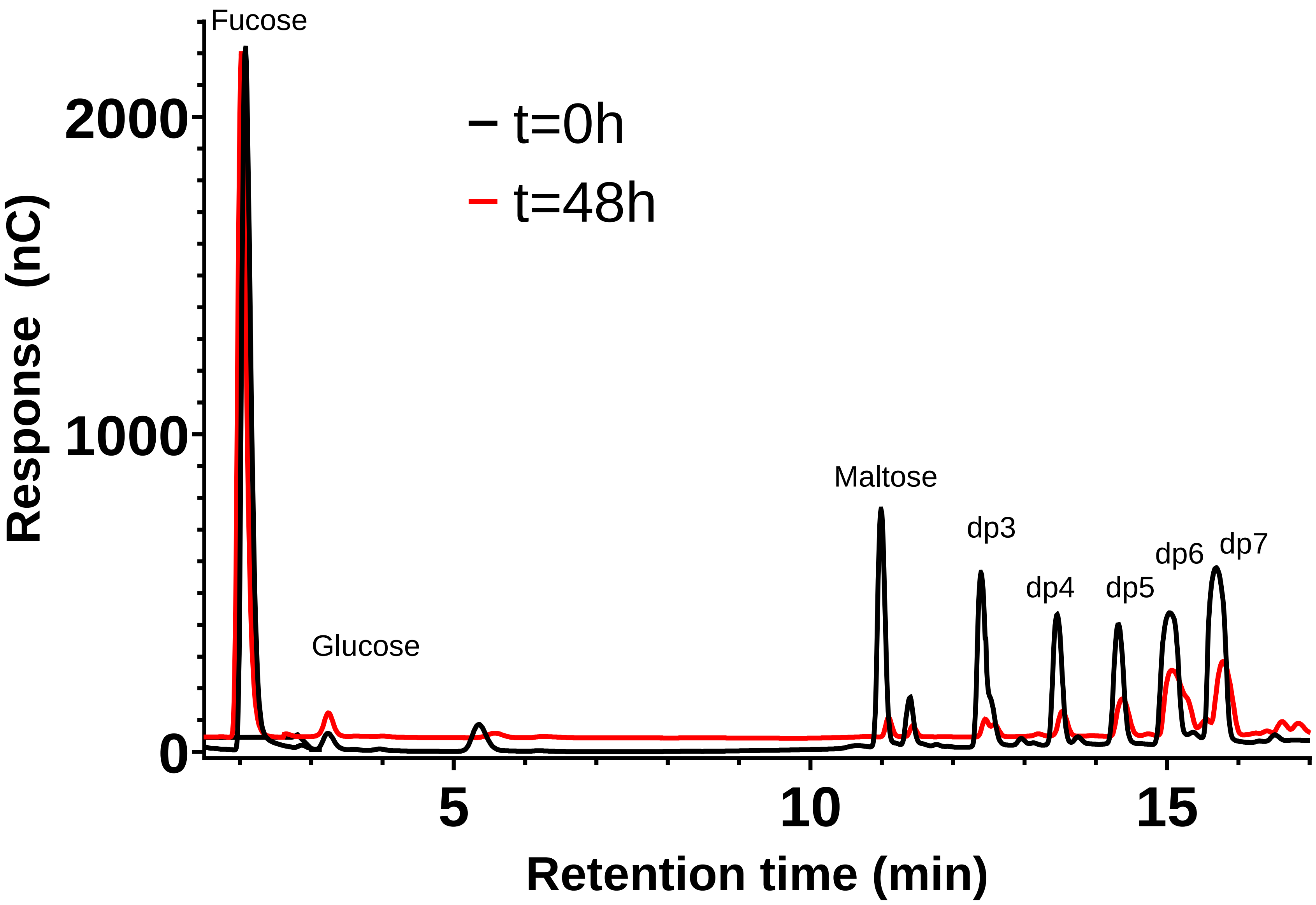

(See legend to **Fig. S1a**)

Figure S1. Degradation and modification of carbohydrates by the three amylases analyzed by Pulsed Amperometric Detection. Enzymes and carbohydrate substrates indicated in the title Transglycosylation was carried out with maltoheptaose and either glucose or fucose.

Figure S2

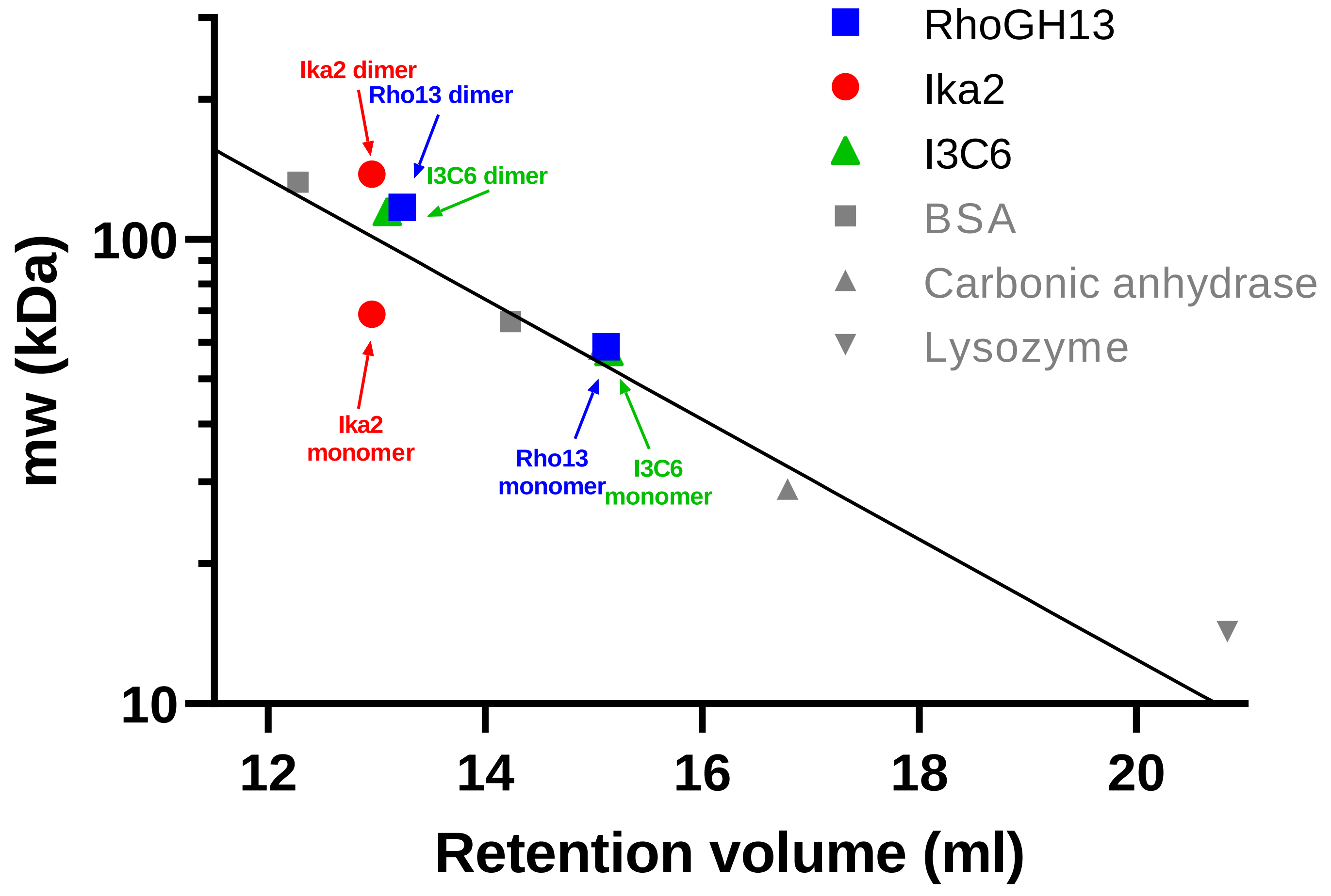

Figure S2. Calibration of the Superdex-200 Increase column used for size analysis of the amylases.

Figure S3ab

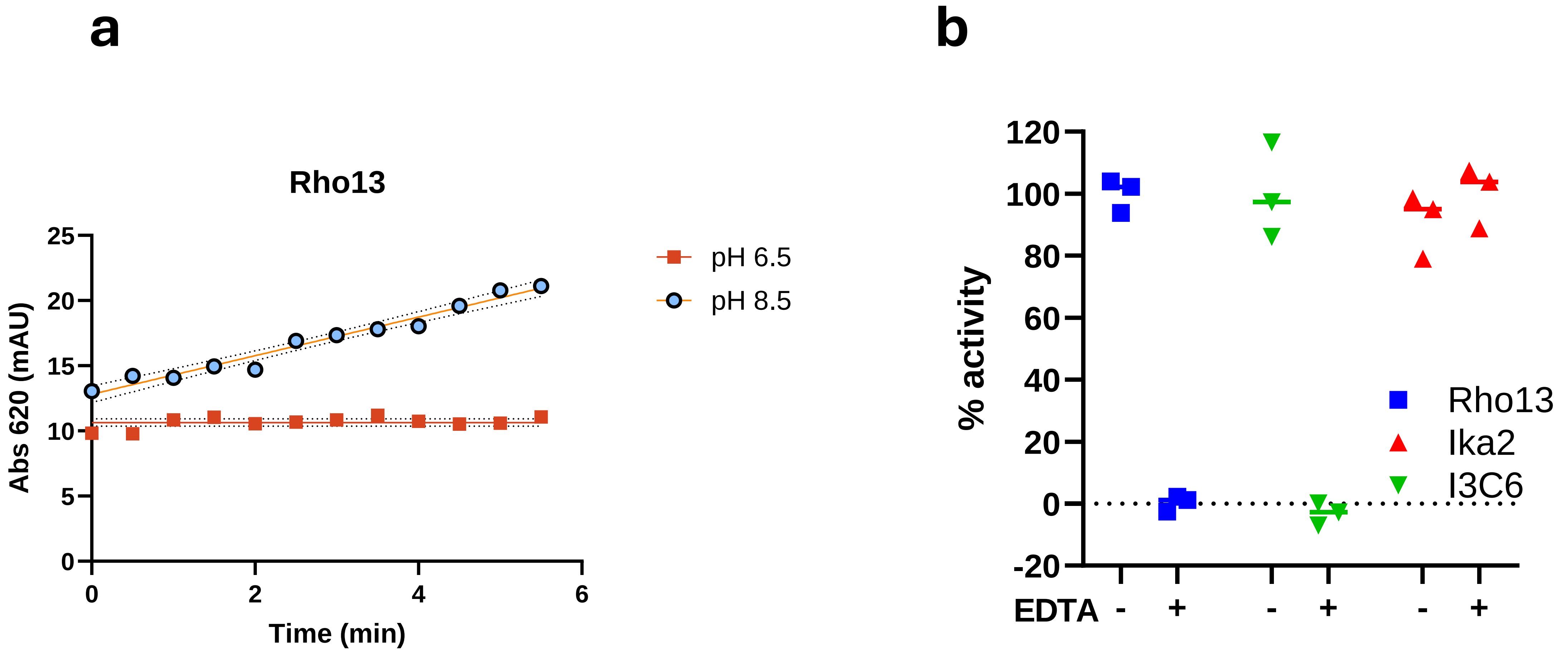

Figure S3. Examples of activity raw data. a) example of raw data of MBTH assay from the pH assay of Rho13 at pH 6.5 (red square) and 8.5 (blue circle) respectively are measured absorbance, line is fit (used for other figures) dotted line is standard error of fit. b) relative activity of enzymes in 2mM $CaCl_2$ buffer ± 3mM EDTA.

Figure S3c

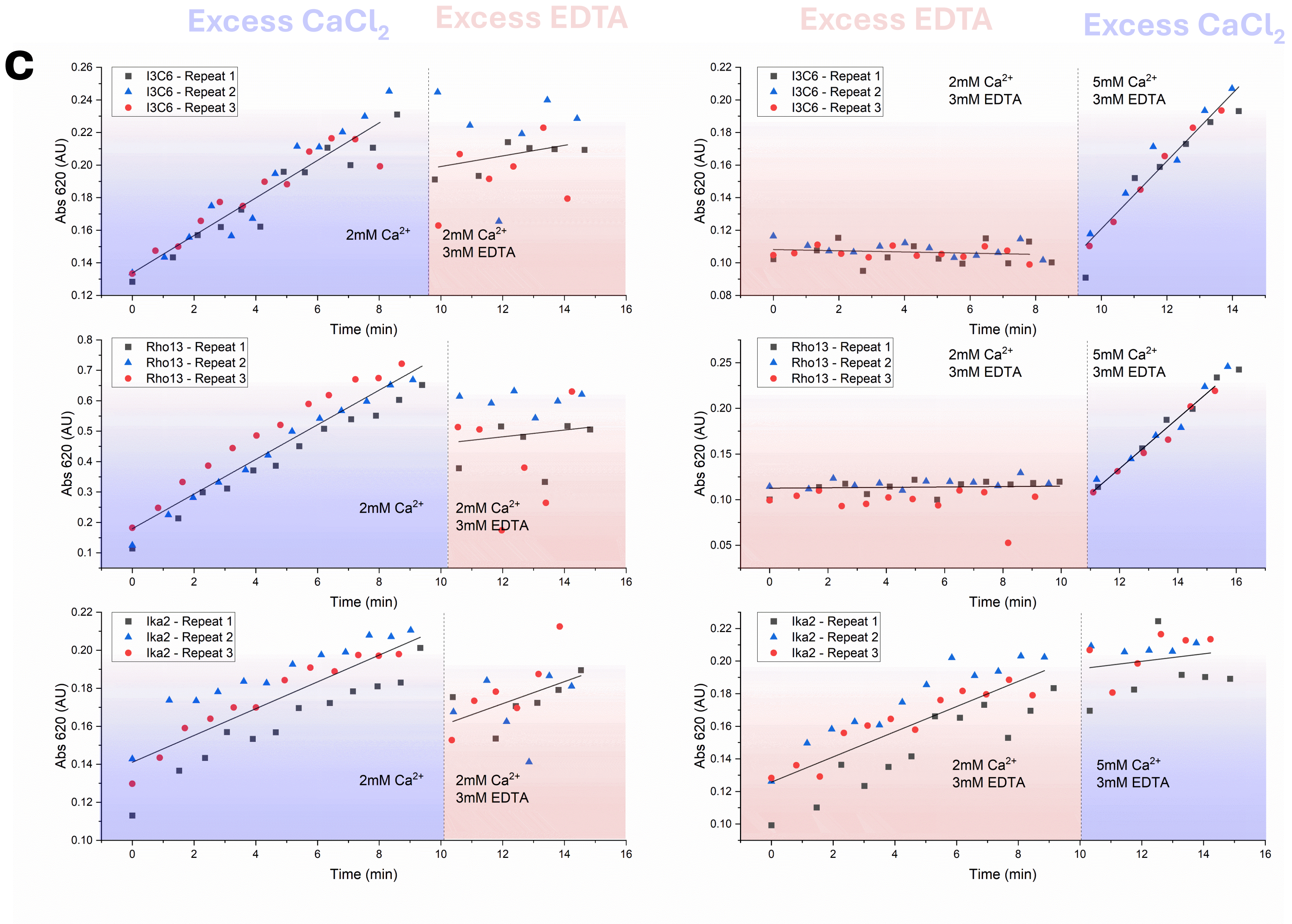

Figure S3. c) MBTH assay in 50mM MOPS buffer pH7.5, 50mM NaCl, 2mM $CaCl_2$ with EDTA added after 10 minutes (left) and in 50mM MOPS buffer pH7.5, 50mM NaCl, 2mM $CaCl_2$, 3mM EDTA with additional 3mM $CaCl_2$ added after 10 minutes (right).

Figure S4

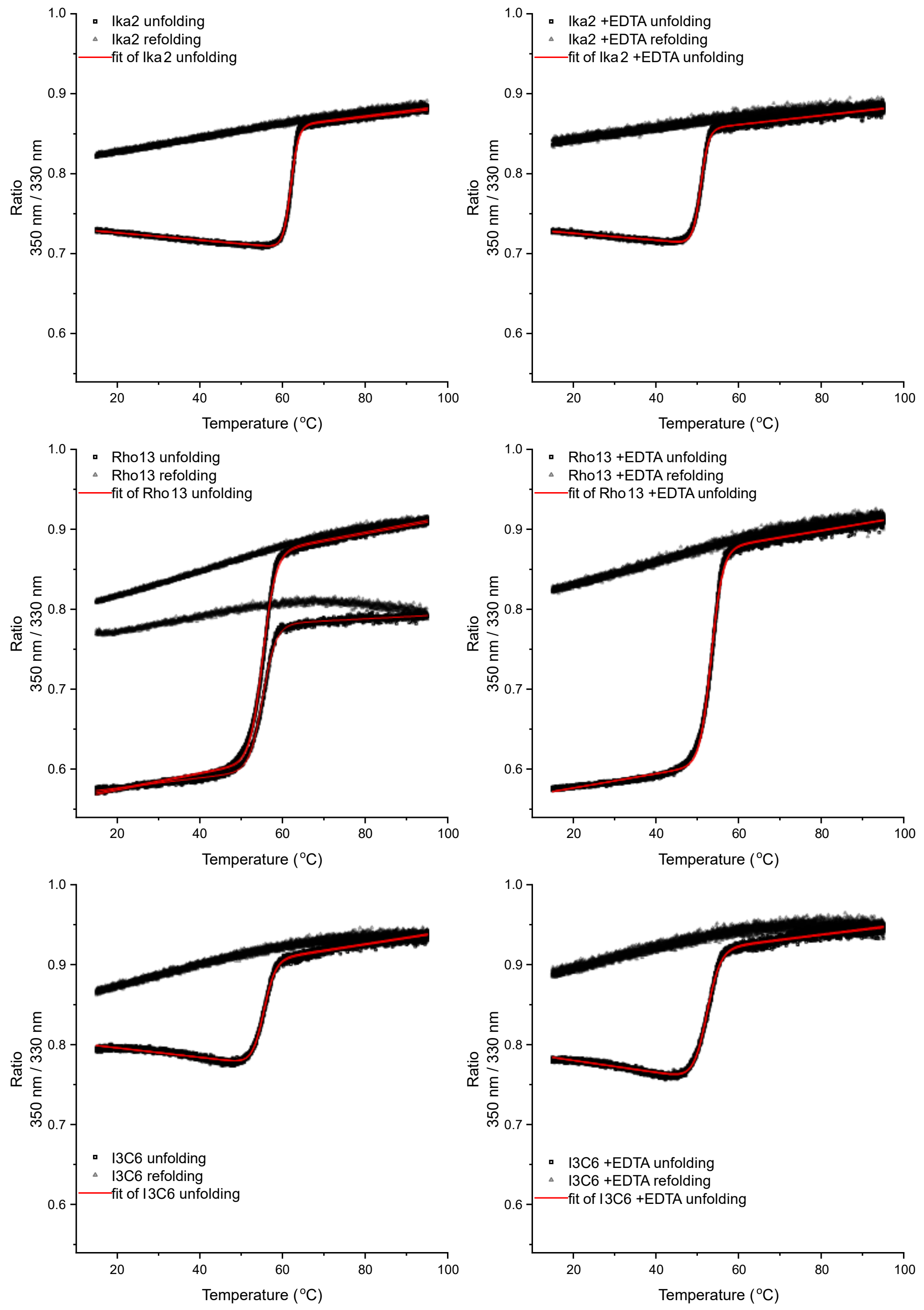

Figure S4. Thermal denaturation of the three enzymes measured by Differential Scanning Fluorometry. All datasets are overlaid replicate data (n=3) of technical repeats in 50mM MOPS buffer pH7.5, 50mM NaCl, 2mM $CaCl_2$ ± 3mM EDTA. Black circles represent the heating/unfolding regime, and grey circles represent the cooling/refolding regime, though no refolding was observed. Red lines represent a fit to a two state unfolding model, with a global fit to unfolding enthalpy and t_m between replicates.

Figure S5

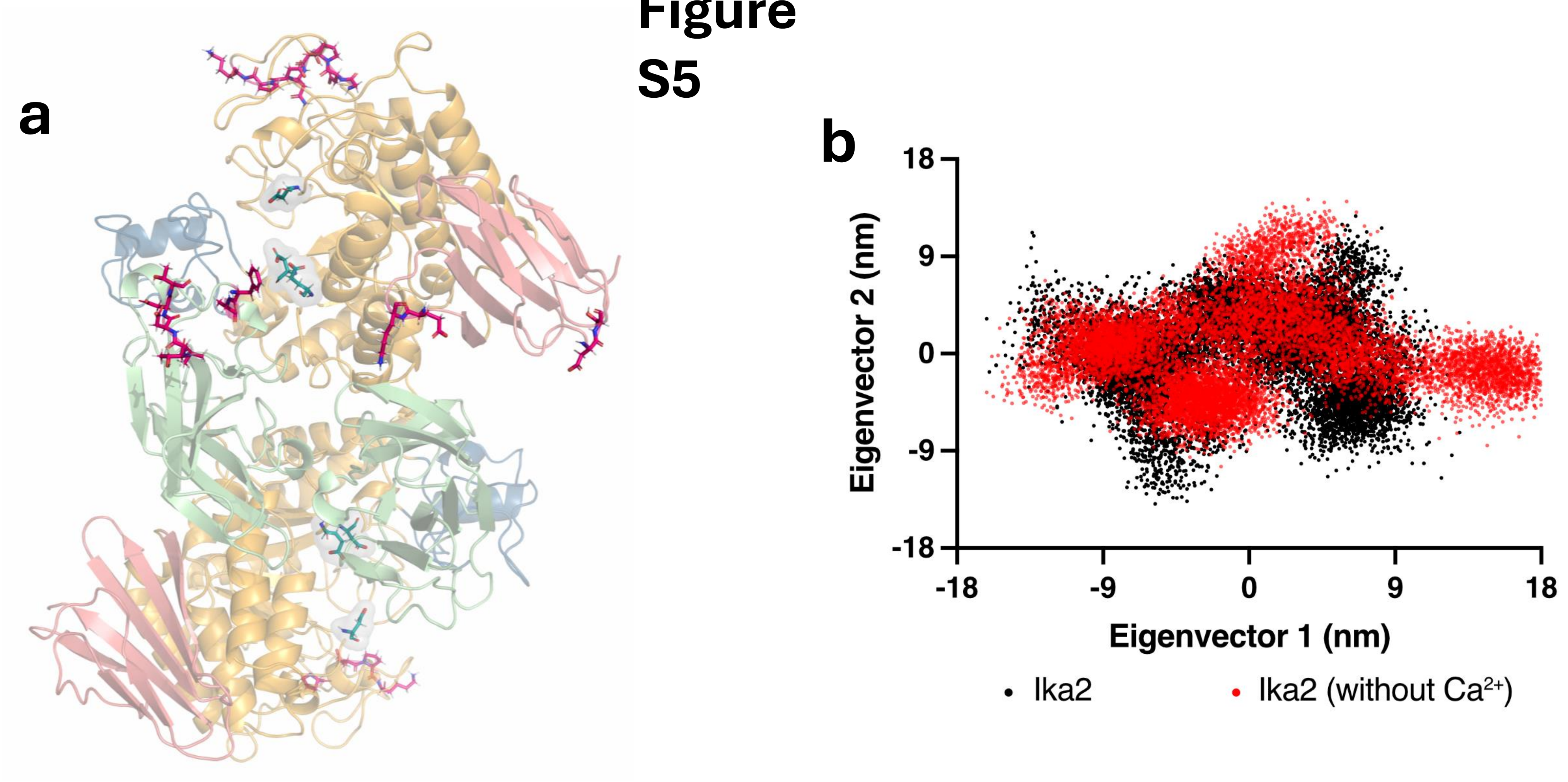

Figure S5. Analysis of the role of Ca^{2+} on structural fluctuations and conformational flexibility in Ika2 (a) Three-dimensional representation of the Ika2 without Ca^{2+} system that highlights the residues that, throughout the 1.2 μs MD simulations, exhibited increased flexibility (represented in magenta sticks) compared to the same enzyme in the presence of calcium. The domains A, B, C, and N are shown in bright orange, iridium blue, salmon, and light green, respectively. The catalytic D330/E359/D426 triad is represented in light blue sticks. (b) Projection of the concatenated 1.2 μs MD trajectories onto the two principal eigenvectors (eigenvectors 1 and 2) for Ika2 with Ca^{2+} (black dots) and Ika2 without Ca^{2+} (red dots).

Figure S6

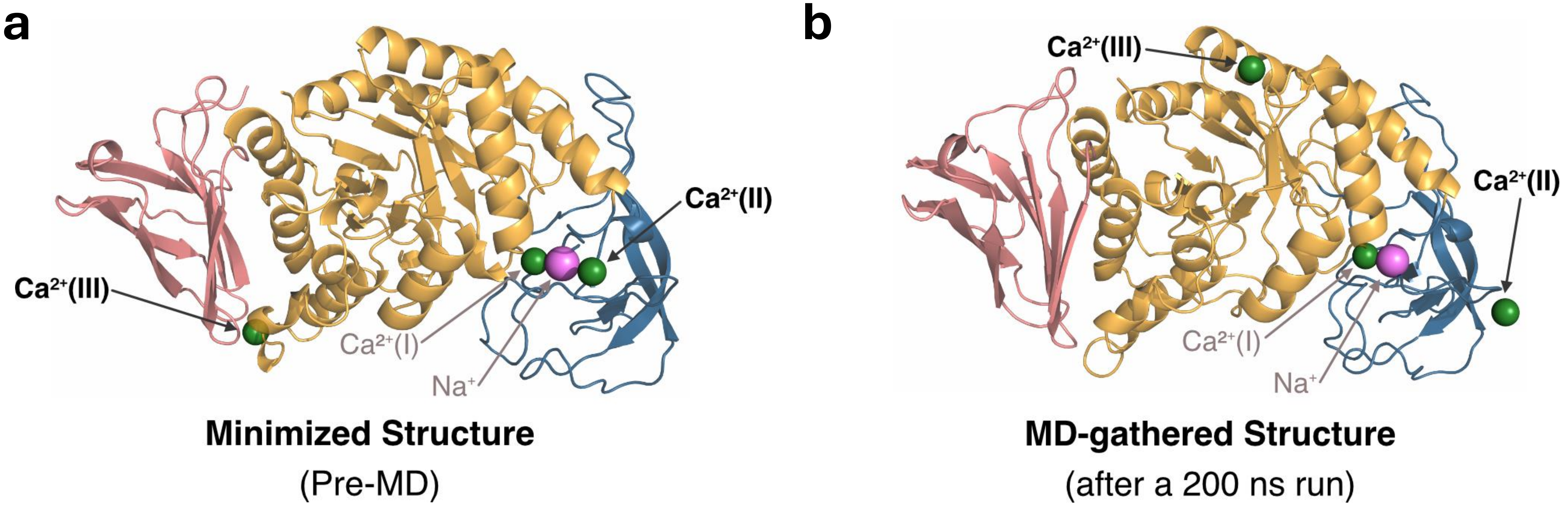

Figure S6. Three-dimensional cartoon representation of the I3C6 enzyme before (a) and after (b) a 200 ns MD simulation. The domains A, B, and C are shown in different colors, and the calcium and sodium ions are depicted as green and violet spheres, respectively. (a) Minimized structure of the I3C6 psychrophilic enzyme, which shows that the Ca^{2+}(I)-Na^{+}-Ca^{2+}(II) linear triad and Ca^{2+}(III) remain lodged in the putative binding sites where they were placed during the modeling procedure. (b) I3C6 structure obtained at the end of a 200 ns MD simulation, which shows both Ca^{2+}(II) and Ca^{2+}(III) abandoned their original binding site during the simulation.

Figure S7

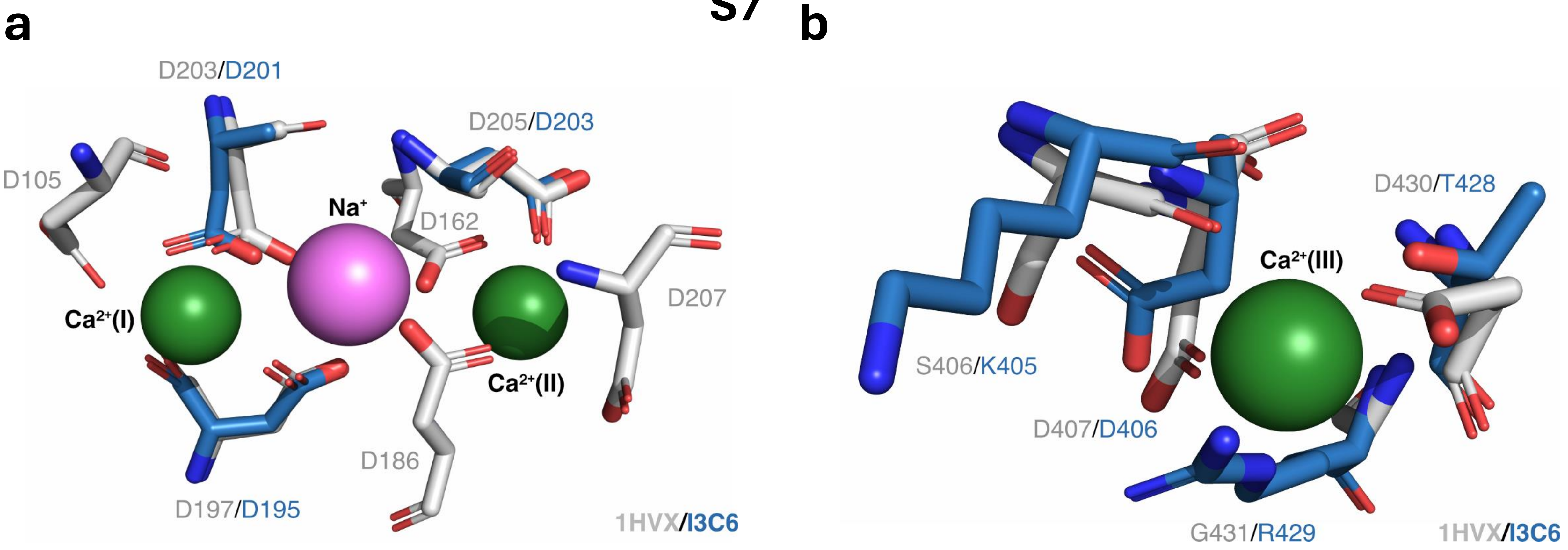

Figure S7. Structural comparison of the amino acid residues involved in metal binding in the I3C6 psychrophilic enzyme (iridium blue sticks) and the 1HVX homolog (white sticks). The calcium and sodium ions are depicted as green and violet spheres, respectively. (a) In the 1HVX structure, seven aspartate residues interact with the Ca^{2+}(I)-Na^{+}-Ca^{2+}(II) linear triad, while only 3 aspartate residues can be found in the same region in the I3C6 structure. (b) In the Ca^{2+}(III) binding site, the 1HVX homolog exhibits a higher count of aspartate residues than the I3C6 enzyme (2 vs. 1). In addition, the psychrophilic system includes two positive residues (K405 and R429) that are not present in the homolog structure.

Figure S8

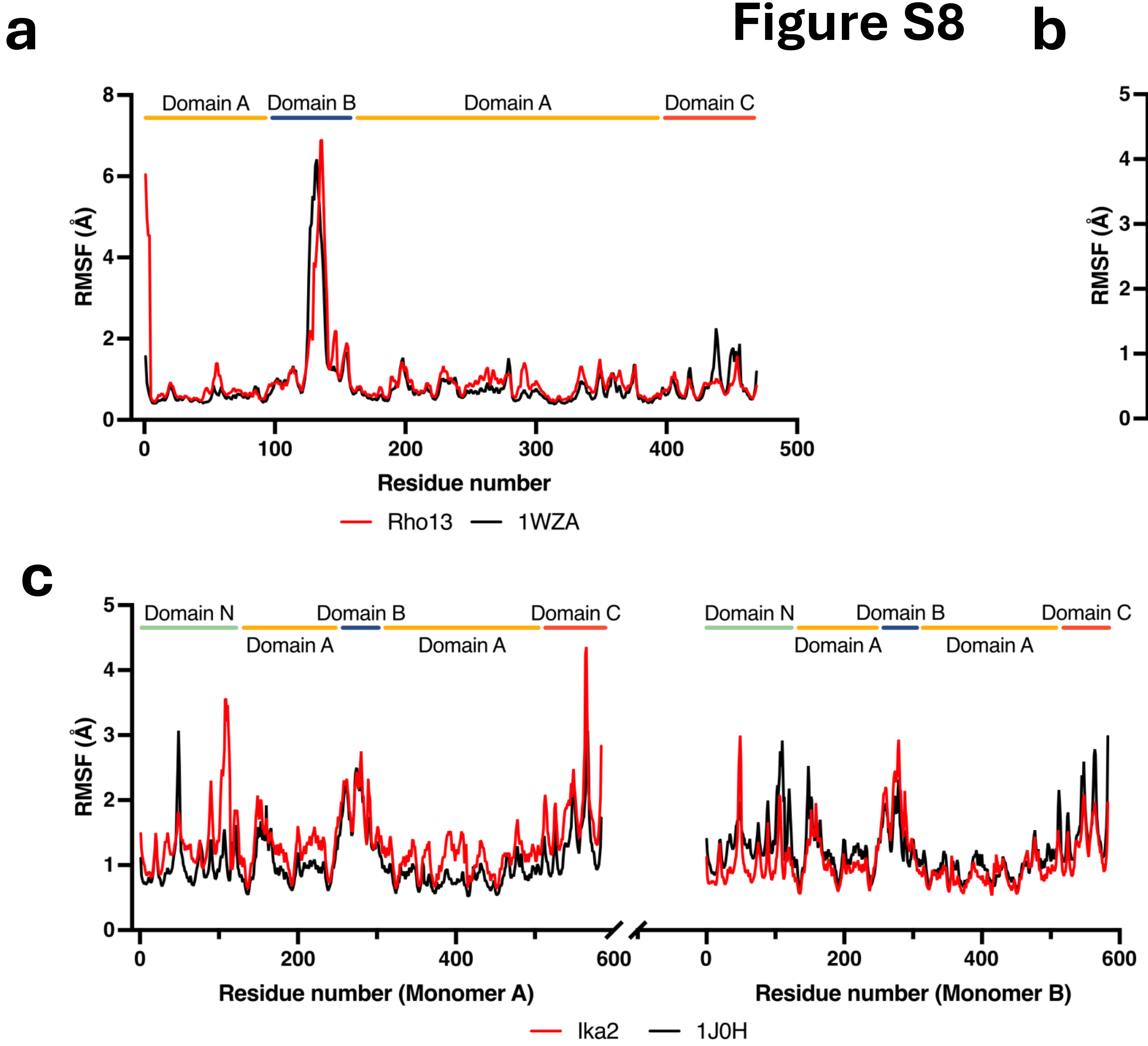

Figure S8. Per-residue RMSF profiles of each pair of psychrophilic (red lines) and homolog (black lines) α-amylases.
